# Acute Effects of Exhaustive Exercise on the Anti-Aging Protein S-Klotho in Young Females and Males with Obesity

**DOI:** 10.3390/jcm15218313

**Published:** 2026-10-26

**Authors:** Michael Svensson, Lisa Blomqvist, Torbjörn Åkerfeldt, Pär Steneberg, Christer Malm

**Affiliations:** 1 Department of Community Medicine and Rehabilitation, Section of Sports Medicine, and Umeå School of Sport Sciences, Umeå University, 901 87 Umeå, Sweden; christer.malm@umu.se; 2 Själevad Health Care Center, Sjukhemsvägen 2 B, 894 30 Örnsköldsvik, Sweden; lisa.blomqvist@rvn.se; 3 Department of Medical Sciences, Clinical Chemistry, Uppsala University, 751 85 Uppsala, Sweden; torbjorn.akerfeldt@akademiska.se; 4 Department of Medical and Translational Biology, Umeå University, 901 87 Umeå, Sweden; par.steneberg@umu.se

**Keywords:** visceral adiposity, systemic inflammation, cardiorespiratory fitness, exerkine, insulin resistance, aging

## Abstract

**Background:** Circulating S-Klotho is increasingly recognized as an anti-aging protein linked to cardiovascular protection and metabolic regulation. Although exercise elevates S-Klotho, the dose–response threshold and metabolic determinants of this response in young adults with obesity, a population at heightened risk for cardiometabolic dysfunction, remain poorly characterized. To examine the acute effects of submaximal and maximal cycling on circulating S-Klotho in young adults with obesity and to identify key metabolic predictors of exercise-induced changes. **Methods:** Eighteen young (26 ± 3 years, BMI 30 ± 2 kg/m^2^) males and females completed incremental cycling to exhaustion. Serum S-Klotho was assessed at rest, after submaximal (SubMax), and post-maximal (Max) exercise. Covariates included cardiorespiratory fitness (VO_2max_), iDXA body composition, HOMA-IR, and hsCRP. Predictor Screening and Partial Least Squares (PLS) regression with fivefold cross-validation were used to identify determinants of exercise-induced S-Klotho responses. **Results:** A significant acute increase in S-Klotho was observed (Main Effect of Time: *F*_(2,32)_ = 9.4, *p* = 0.0006). Post hoc analysis revealed an intensity-dependent threshold, with a significant surge occurring only between submaximal and maximal exertion (*p* = 0.021; Rest to Max: *p* = 0.0005). No significant sex differences or interactions were observed (*p* > 0.05). The PLS model explained 74% of the variance in S-Klotho changes, identifying systemic inflammation (Log_10_[hsCRP]) and visceral adiposity as the primary predictive factors. **Conclusions:** Exhaustive exercise acutely increases circulating S-Klotho in young adults with obesity, independent of sex. This response is intensity-dependent and primarily driven by baseline metabolic risk factors. S-Klotho may represent an acute exercise-responsive biomarker linked to metabolic health in at-risk populations. These exploratory findings warrant confirmation in larger and adequately powered studies.

## 1. Introduction

Obesity exhibits several physiological and cellular hallmarks commonly associated with aging, including disruptions in hormonal homeostasis, insulin sensitivity, amino acid, fatty acid and glucose metabolism, inflammation, autophagy, immune function, mitochondrial function, nutrient sensing, and epigenetic regulation [1,2,3,4]. These alterations may contribute to accelerated biological aging of multiple organs and the vascular system, thereby increasing the risk of obesity-related comorbidities and premature mortality. In this context, α-Klotho has emerged as a hormone-like anti-aging factor with potential roles in metabolic regulation, tissue protection, and the maintenance of cellular homeostasis [5]. The Klotho gene (*KL*), first characterized by Kuro-o et al. in 1997, was shown to cause premature aging and reduced lifespan in transgenic mice when mutated [6]. Subsequent studies demonstrated that restoration or overexpression of Klotho can reverse aging-related phenotypes and extend lifespan in deficient mice [7,8,9]. Highly conserved across species, the Klotho family comprises three paralogues, α-, β-, and γ-Klotho, which contribute to the regulation of metabolic and mineral homeostasis. Among these, α-Klotho, encoded by the *KL* gene, is the most extensively studied and is expressed predominantly in the kidneys, although expression has also been detected in skeletal muscle and other tissues [6,10,11,12,13,14]. Despite the relatively low expression of *KL* mRNA and protein in skeletal muscle [6,12], the substantial mass of this tissue, accounting for approximately 30–40% of total body weight, raises the possibility that skeletal muscle contributes to circulating α-Klotho (S-Klotho) concentrations. S-Klotho is generated primarily through proteolytic shedding of the extracellular domain of membrane-bound α-Klotho. Functionally, S-Klotho serves as an essential co-receptor for Fibroblast Growth Factor 23 (FGF23), regulating phosphates and calcium homeostasis, while also exerting endocrine effects on vascular function, insulin sensitivity, and metabolic health [15].

An increasing body of evidence suggests that low S-Klotho levels have been linked to prediabetes [16,17,18], endothelial dysfunction, and cardiovascular disease [19,20,21]. Individuals with type 2 diabetes (T2D) generally exhibit lower S-Klotho levels than healthy individuals [22,23], whereas higher circulating S-Klotho concentrations appear protective against future coronary artery disease and cerebrovascular events in patients with T2D [24].

Furthermore, S-Klotho is also implicated in chronic kidney disease (CKD), where lower levels are associated with disease progression and poorer prognosis [25,26,27]. A recent meta-analysis demonstrated that exercise interventions can increase circulating S-Klotho levels while simultaneously improving cardiorespiratory fitness and vascular function in individuals with CKD [28]. Additionally, evidence supporting the metabolic relevance of S-Klotho comes from large population-based studies. Low circulating S-Klotho levels have been associated with both the incidence of metabolic syndrome (MetS) and increased mortality risk [29,30]. Furthermore, serum S-Klotho exhibits a U-shaped relationship with all-cause mortality, suggesting that both excessively low and high concentrations may be clinically relevant [31]. Data from the NHANES cohort (2007–2012, ~10,000 participants) demonstrated inverse associations between S-Klotho levels and several components of MetS, particularly abdominal obesity and triglyceride (TAG) levels [32]. Similarly, a negative correlation has been reported between S-Klotho and the triglyceride–glucose index (TyG index) [33,34], which is a recognized surrogate marker of insulin resistance and a predictor of T2D and Metabolic dysfunction-Associated Steatotic Liver Disease (MASLD) [35].

Regular physical activity is a cornerstone in the primary and secondary prevention of cardiovascular and metabolic disorders, demonstrating a dose-dependent reduction in disease incidence and all-cause mortality.

Regular physical activity remains a foundational element in the prevention of cardiovascular and metabolic disorders. Landmark harmonized data from device-measured cohorts underscore a steep, non-linear dose–response relationship, demonstrating that accumulating even modest amounts of daily movement drastically mitigates both cardiometabolic risk and all-cause mortality [36,37,38]. Recent studies suggest a positive association between exercise and systemic S-Klotho levels [39], supporting the notion that S-Klotho may mediate some of the beneficial effects of physical activity as an exerkine [40]. Despite these observations, the precise cellular mechanisms linking exercise-induced changes in S-Klotho to metabolic health remain largely unresolved.

Acute increases in S-Klotho following exercise have been observed [39,40], but studies involving young adults with overweight and obesity remain limited. Given that obesity is a major driver of insulin resistance, T2D, and CVD, determining how exercise regulates circulating S-Klotho in this population may provide important insights into the interplay between metabolic health and biological aging. Such knowledge could help establish S-Klotho as a dynamic biomarker of exercise responsiveness and cardiometabolic health, while informing early lifestyle interventions aimed at preventing progression toward metabolic disease.

### Study Objective

This exploratory study aimed to investigate the acute effects of different stages of incremental ergometer cycling exercise culminating in exhaustion on circulating S-Klotho concentrations in young men and women with overweight or obesity. Additionally, we examined whether sex influences the S-Klotho response and identified metabolic determinants of exercise-induced changes in S-Klotho, including measures of body composition, cardiorespiratory fitness, insulin sensitivity, and systemic inflammation. Based on previous evidence linking low S-Klotho concentrations to adverse cardiometabolic health, we hypothesized that exercise would acutely increase circulating S-Klotho concentrations and that the magnitude of this response would be influenced by underlying metabolic characteristics, particularly adiposity, insulin resistance, and systemic inflammation. By addressing these aims, we sought to advance understanding of the exercise/S-Klotho axis and evaluate the potential of S-Klotho as a dynamic exerkine and biomarker of exercise responsiveness, metabolic resilience, and healthy biological aging.

## 2. Material and Methods

### 2.1. Study Design and Subjects

The present study extends the work of a cohort study that has previously investigated the role of the Paraoxonase 1 (PON1) enzyme in HDL functionality, insulin sensitivity, and muscle metabolism [41], as well as gene expression pathways in human skeletal muscle and adipose tissue [42]. These reports analyzed key covariates, including glucose, insulin, blood lipids, hsCRP, body composition, and maximal oxygen uptake (VO_2max_) in relation to PON1 [41] and transcriptional communities [42].

In the present study, these registered covariates are utilized to examine their relationship with S-Klotho. Details regarding participant recruitment, as well as inclusion and exclusion criteria, have been described previously [41]. In brief, nine men and nine women with overweight or obesity (26 ± 3 years, BMI 30 ± 2 kg·m^−2^), all free from medical diagnoses, were included in this study. Detailed participant characteristics are summarized in Table 1.

All participants provided written and oral informed consent before inclusion and were instructed to avoid exhaustive physical activity for two days prior to this study. The study protocol was conducted in accordance with the Declaration of Helsinki and was approved by the Regional Ethical Review Board in Umeå, Sweden (DNR 13/262-31).

### 2.2. Anthropometrics

Assessment of body weight was conducted using a digital scale (Avery Berkel HL120, Avery Berkel, Smethwick, UK), and body mass index (BMI) was calculated. In addition, measures of fat and lean mass were obtained through Intelligent Dual-energy X-ray Absorptiometry (iDXA, GE Healthcare, Madison, WI, USA).

### 2.3. Blood Sampling and Blood Analyses

For baseline clinical measurements, blood samples were collected in the morning after an overnight fast via venipuncture of the antecubital vein, and handled according to standard procedures. The samples were centrifuged (10 min at 3000× *g*, +4 °C) and serum was stored at −80 °C until routine analyses of hsCRP, glucose, insulin, and blood lipids were conducted at the accredited Clinical Chemistry Department of Uppsala University Hospital, Sweden. The Homeostatic Model Assessment for Insulin Resistance (HOMA-IR) was calculated using the following formula: (fasting insulin [mU·L^−1^] × fasting glucose [mmol·L^−1^])/22.5.

**Figure 1:**
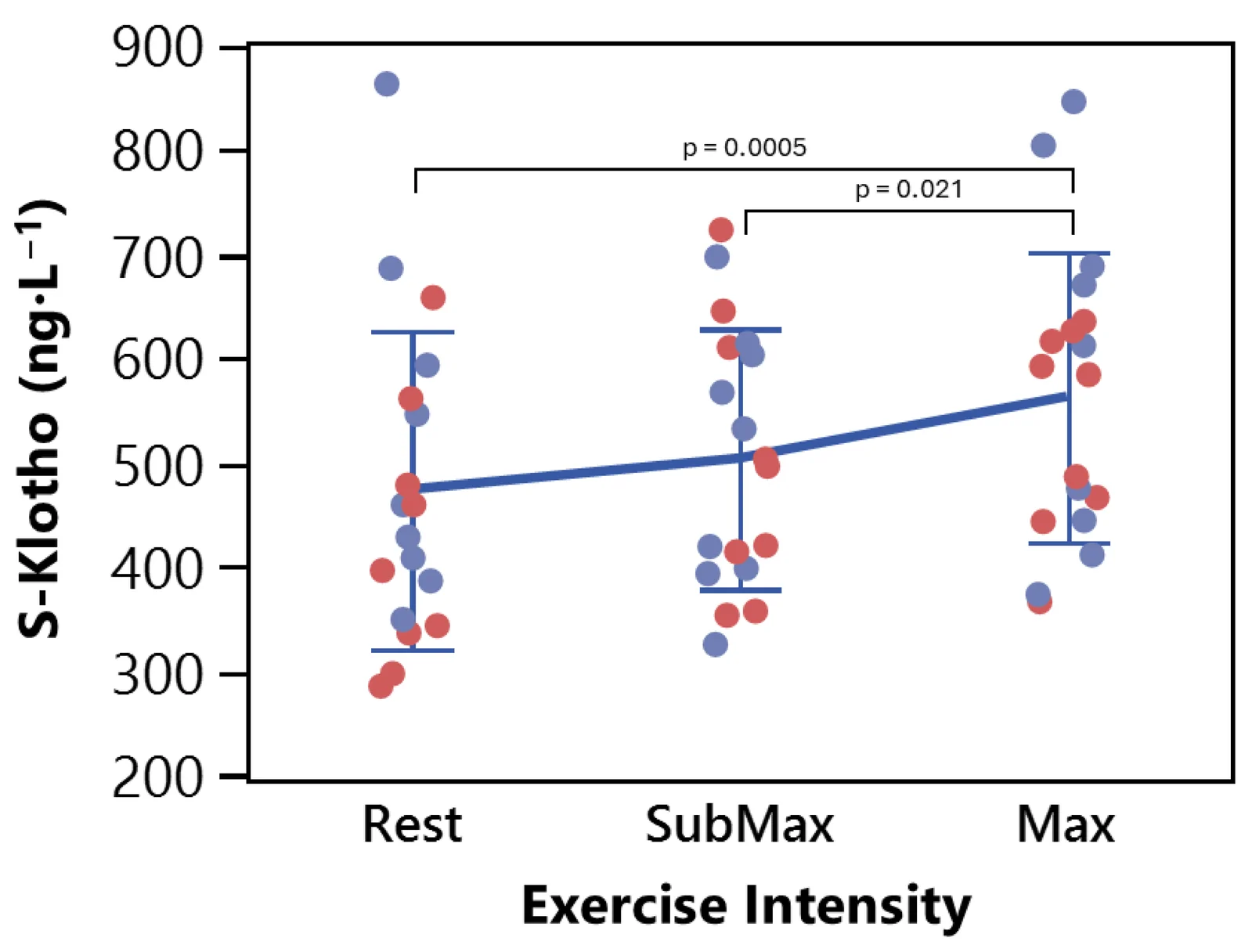
Individual serum S-Klotho concentrations measured at rest, following completion of the submaximal cycling protocol (SubMax), and immediately after maximal exercise to volitional exhaustion (Max) in young adults with overweight or obesity (N = 18). Blue (females, *n* = 9) and red (males, *n* = 9) symbols represent individual participants. Horizontal bars indicate group means ±SD. A significant main effect of time was observed (mixed-effects model, F(2,32) = 9.4, *p* = 0.0006, *F*(2,32) = 9.4, *p* = 0.0006, ηp^2^ = 0.37), with post hoc analysis revealing a significant surge in S-Klotho from Rest to Max (*p* = 0.0005, Hedges’ g = 0.92) and from SubMax to Max (*p* = 0.021), indicating an exercise intensity-dependent response.

For S-Klotho analyses in response to exercise, resting serum blood samples were collected approximately two hours after lunch to minimize the impact of postprandial or circadian fluctuations, followed by samples after submaximal ergometer cycling (SubMax), and immediately following maximal cycling (Max) (Table 2, Figure 1).

### 2.4. Cardiometabolic Fitness

Following a 5 min warm-up at 30–50 W, a submaximal incremental cycling test was performed on a Monark 894 ergometer (Monark Exercise ABB, Sweden), consisting of five incremental workloads, with a starting intensity at 30 W and a continuous increase of 15 W every fourth minute until the 90 W level was completed (pedaling rate 70 rpm). Each four-minute submaximal interval was followed by one minute of recovery, sitting on the ergometer cycle, before the next interval began. After one minute recovery following the fifth workload, the second part of the cycle test to determine VO_2max_ was initiated. The maximal cycling test started at 105 W with an increase of 15 W each minute until exhaustion. The participants were allowed to choose the pedaling rate. VO_2max_ was determined as the highest mean VO_2_ of a continuous 60 s period. Analysis of airflow and respiratory gases O_2_ and CO_2_ was performed with a Jaeger Oxycon Pro system (Erich Jaeger GmbH, Hoechberg, Germany).

### 2.5. S-Klotho and Biochemical Analysis

Assessment of Klotho concentration in serum samples was conducted with the use of an IBL Human soluble alfa-Klotho ELISA Assay Kit (Immuno-Biological Laboratories Co., Ltd., Gunma, Japan), with an intra-assay CV of <5% [43] according to the manufacturer’s protocol.

**Table 2:** Serum Klotho concentration in response to exercise.

	Women, *n* 9			Men, *n* 9		
**Pre Exercise** **, ng·L^−1^**	524 (167)	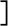	*p* = 0.062	424 (127)	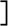	*p* = 0.015
**Post Exercise** **, ng·L^−1^**	591 (174)			535 (97)		

Mean (SD). Paired *t*-test. Reference range 5th and 95th Percentile (Mean ± SD): Women 385–1751 pg·mL^−1^ (874 ± 485), Men 323–1542 pg·mL^−1^ (728 ± 412). From: Espuch-Oliver et al. [44].

## 3. Statistical Analysis

Data normality was assessed using the Shapiro–Wilk Goodness-of-fit test (α < 0.05). Skewed variables, including hsCRP, were Log_10_-transformed prior to analysis. Participant characteristics were compared between sexes using unpaired *t*-tests or Mann–Whitney U tests as appropriate. Differences in absolute S-Klotho concentrations across exercise intensities and between sexes were analyzed using a mixed-effects model fitted with Restricted Maximum Likelihood (REML). ‘Time’, ‘Sex’, and the ‘Time × Sex’ interaction were entered as fixed effects, while participant ‘ID’ was included as a random effect (random intercept) to appropriately account for baseline inter-individual variability and the repeated-measures design. Post hoc pairwise comparisons for significant main effects were conducted using Tukey’s Honest Significant Difference (HSD) test. Observed effect sizes were quantified using partial eta-squared ($\eta_{p}^{2}$) for mixed-effects main effects and Hedges’ g for post hoc pairwise comparisons. Post hoc sensitivity power calculations were performed using G*Power (v3.1.9.7).

Predictive Modeling and Variable Selection: To identify predictors of the exercise-induced change in S-Klotho (Δ S-Klotho) while minimizing the risk of overfitting in a small cohort (N = 18), a multi-step predictive modeling workflow was employed. Candidate predictor variables entered into the modeling procedure included age, sex, BMI, total fat mass, visceral fat mass, lean body mass, waist circumference, VO_2_max, fasting glucose, fasting insulin, HOMA-IR, blood lipids (total cholesterol, LDL-C, HDL-C, and triglycerides), and Log_10_-transformed hsCRP. First, a Predictor Screening (Bootstrap Forest, 100 trees) was conducted to rank potential covariates; only variables contributing >5% to the model’s total gain were considered for further analysis (Supplementary Table S1). We then evaluated four distinct modeling frameworks: Fit Least Squares, Generalized Regression (Lasso), Neural Networks, and Partial Least Squares. To ensure model stability, 5-fold cross-validation was used to calculate the Mean Validation R^2^ and Root Average Squared Error (RASE). Models were manually simplified to include the minimum number of predictors required to maintain peak R^2^. To ensure transparency in our predictive modeling workflow, detailed diagnostic reports, including cross-validation folds and standardized coefficients for the Bootstrap Forest, GLM Lasso, and PLS models, are provided in the Supplementary Table S1 and File S1 to S6.

Statistical analyses were performed using JMP^®^ Pro Edition 19.0.1 (JMP Statistical Discovery LLC, Cary, NC, USA).

## 4. Results

Participant characteristics are presented in Table 1. Men exhibited significantly higher lean body mass and VO_2_max compared to women, whereas indices of adiposity and fasting blood markers did not differ significantly between sexes (Table 1).

### 4.1. Exercise-Induced Changes in S-Klotho

Our study indicates significant acute changes in absolute S-Klotho levels across the different exercise intensities (mixed-effects model, Main Effect of Time: F_(2,32)_ = 9.4, *p* = 0.0006, Table 2, Figure 1). Variance component analysis revealed that inter-individual baseline differences accounted for approximately 80% of the total variance in S-Klotho levels. There was no significant difference in the overall response between men and women (Main Effect of Sex: F_(1,16)_ = 0.76, *p* = 0.40), and no significant interaction between sex and exercise over time (F_(2,32)_ = 2.7, *p* = 0.08).

Consequently, predictive models for the exercise-induced change in Klotho were not split by sex; instead, sex was evaluated as a potential baseline covariate. Post hoc pairwise comparisons by Tukey’s HSD confirmed a significant intensity-dependent threshold for S-Klotho release. While S-Klotho levels trended upward during submaximal exercise, this initial increase from rest was not statistically significant (*p* = 0.340). However, the subsequent transition from submaximal to maximal exertion induced a significant surge in S-Klotho (*p* = 0.021), culminating in a highly significant overall increase from resting baseline to maximal exercise (*p* = 0.0005).

### 4.2. Predictor Screening and Model Selection

To avoid overfitting and reduce the number of variables, the importance of baseline characteristics was first investigated using a Predictor Screening ranking (Supplementary Table S1 and File S1 to S6). Because Fat Mass and Body Weight are strongly correlated, highly collinear anthropometric variables were filtered. Log_10_[CRP], visceral fat, and waist circumference emerged as the most robust baseline predictors of the exercise-induced change in S-Klotho.

**Figure 2:**
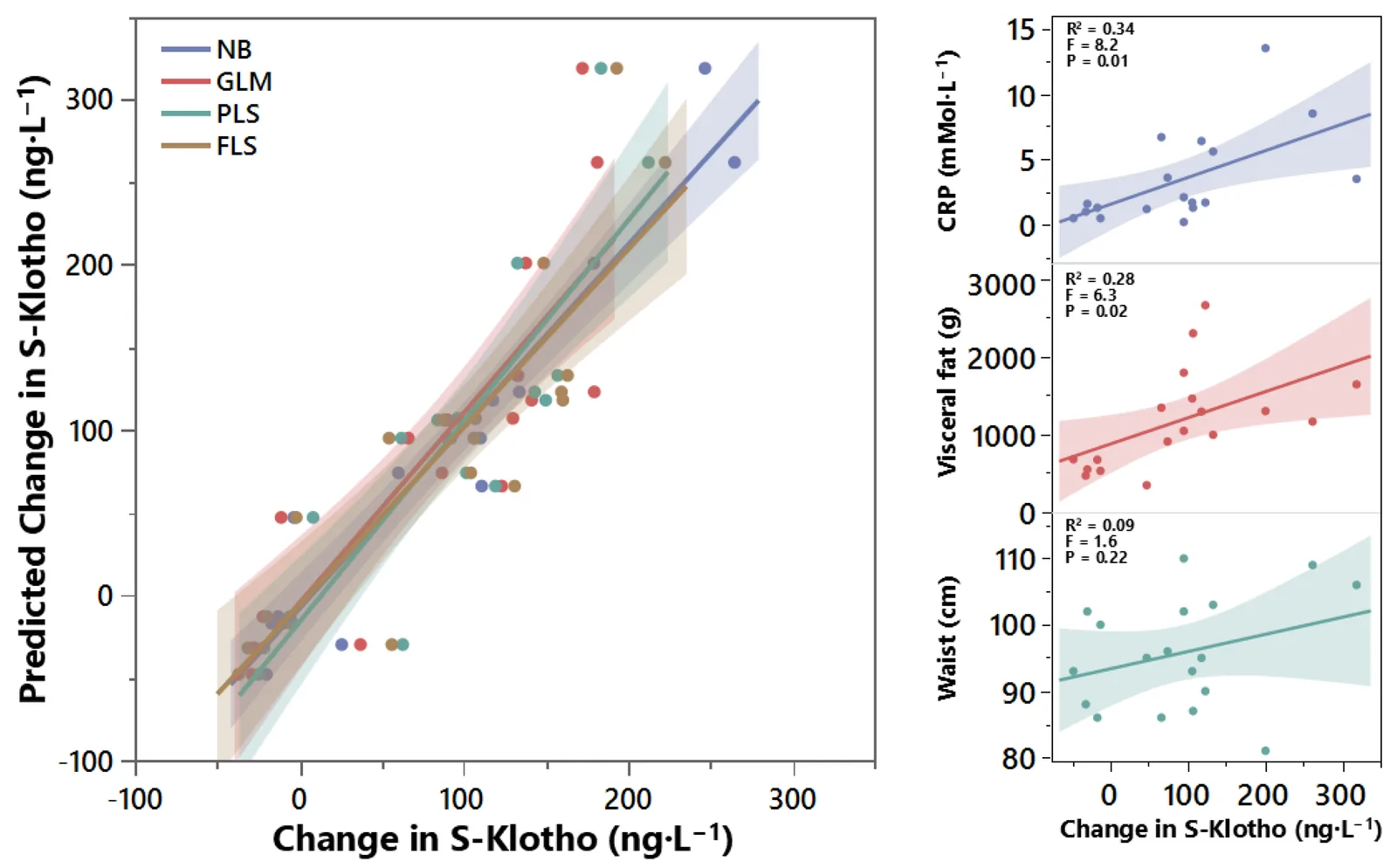
Predictive modeling and univariate correlations for exercise-induced changes in S-Klotho (ΔS-Klotho) in young adults with overweight or obesity (*n* = 18). (Left panel) Predicted versus observed change in serum S-Klotho (ΔS-Klotho) generated using four statistical modeling frameworks: Neural Boosted (NB), Generalized Linear Model Lasso (GLM), Partial Least Squares (PLS), and Fit Least Squares (FLS). Shaded regions represent 95% confidence intervals around model predictions. (Right panels, top to bottom) Univariate associations between ΔS-Klotho and primary cardiometabolic predictors identified during feature screening: Log10-transformed high-sensitivity C-reactive protein (hsCRP; R2 = 0.34, F(1,16) = 8.2, *p* = 0.01), visceral fat mass (R2 = 0.28, F(1,16) = 6.3, *p* = 0.02), and waist circumference (R2 = 0.09, F(1,16) = 1.6, *p* = 0.22). Linear regression lines with 95% confidence bands demonstrate significant positive associations for baseline inflammation (hsCRP) and central adiposity (visceral fat mass), whereas waist circumference was not significantly associated with ΔS-Klotho as a standalone predictor.

Selected variables were entered into multiple predictive modeling frameworks (Table 3). Initial assessment using a Generalized Linear Model (GLM) Lasso and a Neural-Boosted (NB) ensemble showed high training accuracy (R^2^ > 0.76 and R^2^ > 0.87, respectively). However, under rigorous cross-validation, the GLM Lasso demonstrated significant instability, and the NB model was deemed too complex for the sample size, risking severe overfitting. By contrast, the Partial Least Squares (PLS) model achieved the optimal balance of predictive accuracy and stability across validation folds, effectively managing the collinearity between visceral adiposity and systemic inflammation (Table 3; Figure 2, left panel).

### 4.3. Drivers of Exercise-Induced Klotho Changes

Univariate analysis confirmed that both systemic inflammation and adiposity were significant drivers of the acute S-Klotho response (Figure 2, right panels). Specifically, CRP (upper) explained 34% of variance in the change in S-Klotho (F_(1,16)_ = 8.2, *p* = 0.01), while visceral fat mass (middle) explained 28% (F_(1,16)_ = 6.3, *p* = 0.02). Waist circumference (bottom) did not reach statistical significance as a standalone predictor (R^2^ = 0.09, F_(1,16)_ = 1.6, *p* = 0.22). When integrated into a multivariate framework, the PLS model (Figure 2, left panel) provided the most stable predictive performance across cross-validation folds, effectively utilizing the shared variance between these markers to explain 74% of the total exercise-induced change in S-Klotho. Standardized coefficients from the PLS model identified baseline Log_10_[CRP] and visceral fat as the primary drivers of the acute response, confirming that higher adiposity and inflammation at baseline reliably predict larger post-exercise increases in Klotho.

### 4.4. Baseline Klotho, Age, and Mortality Risk Context

**Figure 3:**
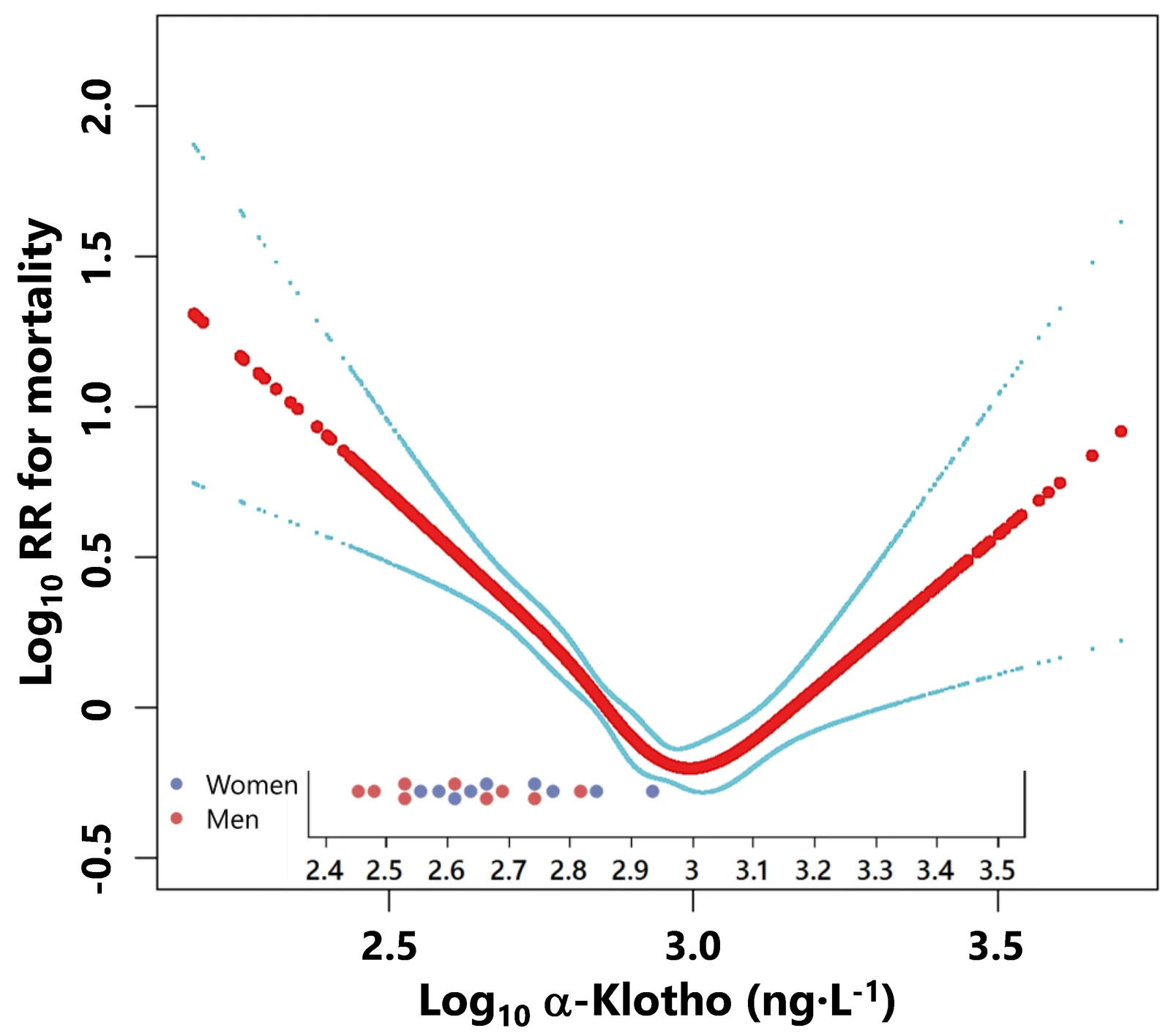
Baseline circulating S-Klotho concentrations contextualized against previously reported mortality risk associations. Individual baseline S-Klotho concentrations from the present cohort (N = 18; blue symbols = females, *n* = 9, red symbols = males, *n* = 9) superimposed on the non-linear, U-shaped association between circulating α-Klotho concentrations and all-cause mortality risk reported by Song et al. [31] The solid curve represents the estimated mortality risk association, with dashed lines indicating 95% confidence intervals. Participants clustered predominantly on the descending left arm of the curve, below the mortality risk nadir (approximately 800–1000 pg·mL^−1^), indicating relatively low baseline S-Klotho concentrations compared with the range associated with the lowest mortality risk in the reference population.

Superimposed data from our present study support the notion that otherwise healthy, young individuals in our cohort possess resting S-Klotho levels positioned on the descending limb of the mortality curve, below the inflection point (nadir, approx. Log_10_ 3.0) where risk is minimized (Figure 3).

Linear regression analysis revealed a significant negative association between participant age and resting S-Klotho levels with F_(1,16)_ = 7.8, *p* = 0.01. Age explained 33% of the variance in baseline Klotho (R^2^ = 0.33), with a notable decline observed from the youngest participants (approx. 850 ng·L^−1^) to those in their early thirties (approx. 350–450 ng·L^−1^).

## 5. Discussion

This study investigated the acute response of circulating S-Klotho to exhaustive exercise in young adults with overweight or obesity, while evaluating the influence of baseline health and body composition covariates. To our knowledge, this is the first investigation to assess the acute effects of maximal exercise on S-Klotho concentrations specifically within this demographic. The primary findings demonstrate that exhaustive exercise induces a significant, intensity-dependent acute surge in S-Klotho. Furthermore, predictive modeling revealed that baseline systemic inflammation (Log_10_[hsCRP]) and visceral adiposity are the most robust primary drivers of this exercise-induced S-Klotho response.

### 5.1. Exercise

With respect to exercise, our findings are consistent with previous research demonstrating an acute rise in S-Klotho following exercise across various populations [45,46,47,48,49]. For example, aerobic exercise has been shown to increase S-Klotho levels, whereas plyometric exercise led to a decrease in two independent groups of men [45]. Similarly, Tarp et al. reported an acute increase in S-Klotho with aerobic exercise in healthy men and women [50], whereas Santos-Dias observed a significant increase only in women [47]. Moreover, Ercan et al. demonstrated that both rheumatoid arthritis patients and healthy controls experienced an acute increase in S-Klotho following endurance exercise, reinforcing Klotho’s responsiveness to physical activity [48].

Discrepancies among existing studies may largely be attributed to differences in exercise protocols, specifically intensity. A review by Avin et al. [51] included unpublished data indicating that S-Klotho levels did not increase following low-intensity aerobic exercise in either young women with obesity or older women. Our data clarify this by revealing an intensity-dependent threshold: while submaximal exercise resulted in a non-significant upward trend, maximal exertion was required to trigger a statistically significant surge. Baseline fitness and age also appear to modulate this response. Saghiv et al. reported that well-trained individuals possess significantly higher baseline S-Klotho levels than untrained counterparts, and that younger untrained adults exhibit higher levels than older untrained individuals [52]. This aligns with observations of elevated resting S-Klotho in male athletes compared to non-athlete controls [53]. While the present findings support the existence of an exercise-intensity threshold for acute S-Klotho release, this study was not designed to distinguish the independent effects of exercise intensity, exercise duration, and total work performed. Although participants completed varying durations of the maximal exercise phase before reaching volitional exhaustion, the relatively small sample size and limited variation in protocol duration preclude firm conclusions regarding the contribution of exercise volume to the observed S-Klotho response. Future studies employing exercise protocols specifically designed to dissociate intensity from duration will be required to determine whether accumulated work, time spent at high intensity, or peak physiological stress is the primary driver of acute S-Klotho release.

### 5.2. Biology

The precise physiological and molecular mechanisms underlying the acute systemic increase in S-Klotho, as well as its tissue of origin during exercise, remain unclear. α-Klotho expression has been demonstrated in several tissues, including the kidney and skeletal muscle [6]. However, because blood samples in the present study were obtained from the systemic circulation, it is not possible to determine whether the observed increase originated from skeletal muscle, kidney, or other tissues.

Considering the complexity of muscle biology and the general decrease in muscle protein synthesis during exercise [54], it is plausible that Klotho previously synthesized in skeletal muscle is stored in vesicles and subsequently released into circulation during exhaustive physical activity, potentially functioning as an exercise-responsive signaling factor, or exerkine. Supporting this hypothesis, Klotho protein expression has been detected in human skeletal muscle localized near the sarcolemma [55]. Given the substantial proportion of muscle mass in the human body, even low muscle-specific expression may potentially contribute to systemic S-Klotho levels. However, this possibility remains speculative, as no published study has directly demonstrated exercise-induced release of S-Klotho from skeletal muscle into the circulation. Likewise, the potential contribution of renal or other tissue sources during exercise has not been directly established.

The acute responsiveness of circulating S-Klotho to exercise suggests the involvement of exercise-sensitive regulatory pathways. However, whether this reflects enhanced proteolytic shedding of membrane-bound Klotho, release from intracellular stores, altered secretion from one or multiple tissues, or a combination of these mechanisms remains unknown. Future studies employing tissue-specific approaches, such as arterio-venous balance measurements, tissue sampling, or molecular tracing techniques, will be required to identify the source and regulation of exercise-induced S-Klotho release. Furthermore, exercise training interventions have been shown to elevate resting systemic S-Klotho levels [40], potentially mediated by concurrent increases in lean mass and reductions in fat mass [30,56]. Nevertheless, the tissue origin and molecular mechanisms responsible for these sustained increases remain incompletely understood.

### 5.3. Cardiometabolic Health and Mortality Risk

Our predictive modeling emphasizes the critical importance of body composition and inflammatory markers in understanding exercise-driven S-Klotho modulation. Specifically, visceral fat mass and hsCRP (both well-established cardiometabolic risk factors) were positively associated with the magnitude of the acute S-Klotho surge. This finding is particularly interesting when contextualized with recent cohort data demonstrating a significant inverse relationship between resting S-Klotho levels and cardiometabolic risk scores in sedentary adults [57], as well as a U-shaped relationship between S-Klotho and all-cause mortality [31].

Because low systemic Klotho levels have been associated with adverse cardiovascular disease (CVD), maintaining optimal Klotho levels is likely crucial for long-term cardiovascular outcomes, Klotho has emerged as a candidate biomarker of cardiovascular and metabolic health. At the molecular level, Klotho supports vascular function by mitigating oxidative stress via the Nrf2 pathway [58,59], exerting anti-inflammatory effects by interfering with NF-κB and TGF-β signaling [60], and promoting endothelial function by stimulating nitric oxide (NO) production [61]. Furthermore, Klotho helps preserve endothelial integrity and regulates blood pressure by modulating calcium and phosphate metabolism. This is highly relevant to chronic kidney disease (CKD), a global health concern amplified by aging, obesity, and type 2 diabetes [62], which is inherently characterized by Klotho deficiency. In CKD, hyperphosphatemia worsens systemic inflammation and vascular calcification, a major predictor of cardiovascular mortality [63,64]. Consequently, Klotho has emerged as a critical biomarker and potential therapeutic target for both CKD and CVD [65,66].

**Figure 4:**
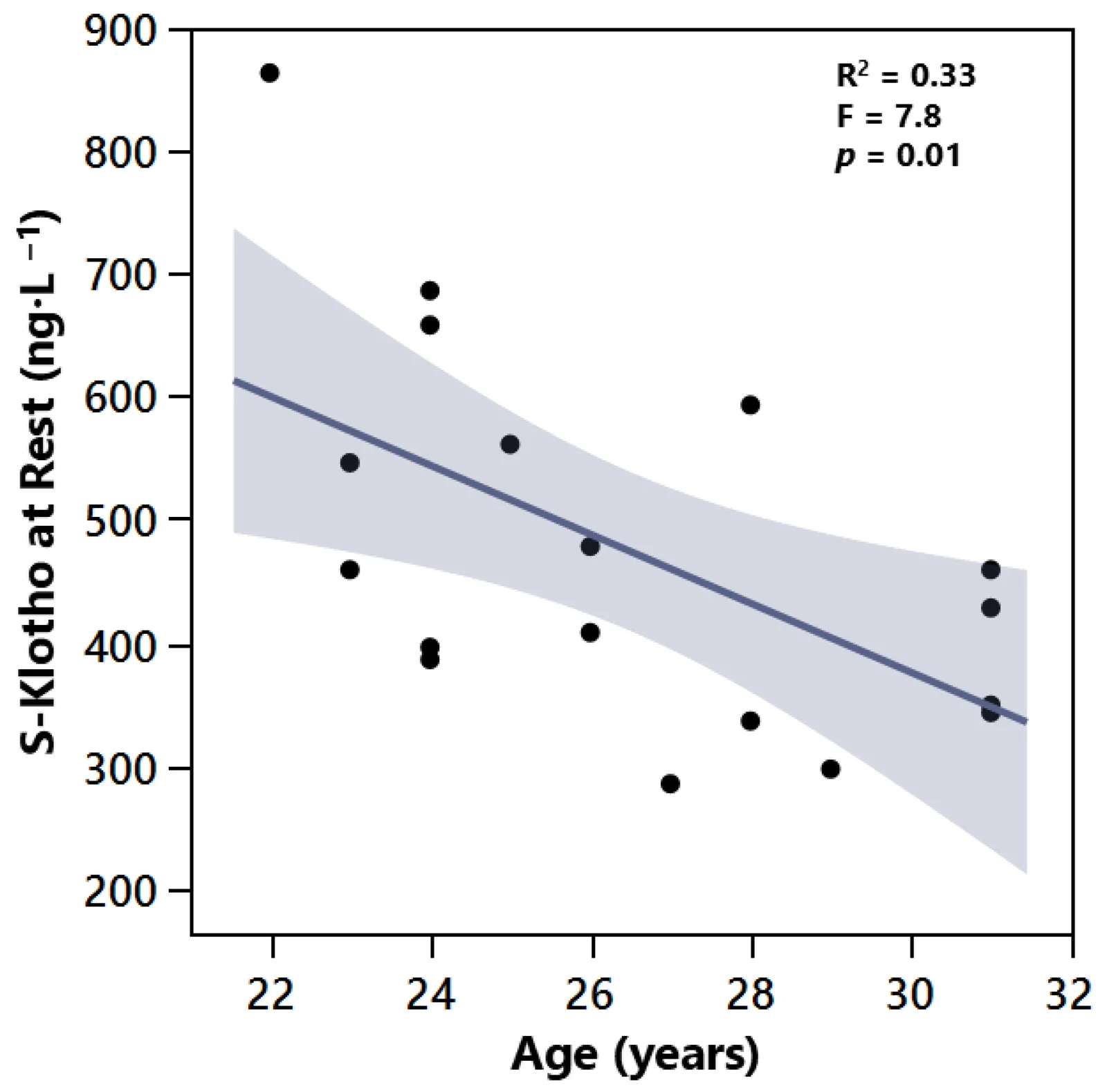
Linear association between age and baseline resting serum S-Klotho concentrations in young adults with overweight or obesity (n = 18). Linear regression line with 95% confidence interval (shaded region) demonstrates a significant negative association between participant age and resting S-Klotho concentrations (R2 = 0.33, F(1,16) = 7.8, *p* = 0.01). Increasing age explained 33% of the variance in baseline S-Klotho, showing a marked decline from early adulthood (approx. 850 ng L^−1^ at 22 years of age) to the early thirties (approx. 350–450 ng L^−1^).

Consistent with the age-related decline in Klotho expression previously reported in the literature, our cohort demonstrated a significant negative correlation between age and resting S-Klotho (R^2^ = 0.33, *p* = 0.01; Figure 4). When contextualized against mortality risk curves [31], these findings suggest that as individuals age, their resting Klotho levels naturally migrate away from the mortality risk nadir (below the inflection point) and toward the ascending limb of the risk curve (Figure 3).

The acute increase in systemic S-Klotho observed following exhaustive ergometer cycling demonstrates that S-Klotho is responsive to acute physiological stress. However, the biological significance of the observed increase remains uncertain. Although higher resting S-Klotho concentrations have been associated with favorable health outcomes, it is currently unknown whether a transient exercise-induced increase translates into meaningful long-term physiological or clinical benefits Future longitudinal studies are therefore required to determine whether repeated exercise-induced elevations in S-Klotho contribute to chronic adaptations in metabolic and cardiovascular health.

## 6. Conclusions

This study demonstrates that acute exhaustive exercise significantly increases circulating S-Klotho levels in young adults with overweight or obesity. An intensity-dependent threshold was identified, whereby maximal exertion, but not submaximal exercise, elicited a significant increase in S-Klotho. Furthermore, visceral adiposity and systemic inflammation emerged as the strongest predictors of the exercise-induced response. Although the physiological mechanisms and tissue source underlying this increase remain unclear, these findings demonstrate that circulating S-Klotho is responsive to high-intensity exercise and may provide a useful model for investigating interactions between exercise, metabolic health, inflammation, and biological aging. These exploratory findings warrant confirmation in larger and adequately powered studies.

## 7. Implications

Physiological implications. Our findings suggest that individuals with greater visceral adiposity and systemic inflammation exhibit a more pronounced acute S-Klotho response to maximal exercise. One possible interpretation is that metabolic status influences the regulation of S-Klotho during acute physiological stress. The observation that visceral adiposity and hsCRP emerged as the strongest predictors of the S-Klotho response suggests a specific relationship between cardiometabolic risk factors and exercise-induced S-Klotho regulation.

Clinical implications. The observed associations between visceral adiposity, systemic inflammation, and exercise-induced changes in S-Klotho indicate that these factors may modify S-Klotho responsiveness to exercise. However, given the exploratory nature of this study and its modest sample size, these findings should be interpreted with caution and require validation in larger cohorts. While the PLS model explained 74% of the variance in the S-Klotho response, the underlying biological mechanisms remain incompletely understood.

In summary, visceral adiposity and systemic inflammation appear to influence the magnitude of the acute S-Klotho response to exercise, highlighting a potential link between metabolic risk and exercise-induced S-Klotho regulation. Future studies are warranted to determine whether targeting these factors enhances long-term S-Klotho regulation and exercise adaptation. Furthermore, identifying the tissue source(s) responsible for exercise-induced S-Klotho release, including the potential contribution of exercising skeletal muscle, remains a key unresolved question in Klotho biology and will require tissue-specific approaches such as arterio-venous balance measurements across skeletal muscle during exercise.

## 8. Limitations

Several limitations should be acknowledged. First, this was an exploratory study with a relatively small sample size (N = 18). Although this study was sufficiently powered to detect the primary effect of exercise intensity on circulating S-Klotho concentrations, it was likely underpowered to detect more subtle sex-specific effects. Second, although significant exercise-induced increases in S-Klotho were observed, the magnitude of change was modest relative to the between-subject variability in resting concentrations. Accordingly, the physiological and clinical significance of these acute changes remains uncertain. Third, the absence of a normal-weight control group precludes direct conclusions regarding obesity-specific S-Klotho responses. Finally, because blood samples were obtained from the systemic circulation, the tissue source of the exercise-induced increase in S-Klotho could not be determined. Future studies involving larger cohorts, appropriate control groups, and longitudinal exercise interventions are needed to establish the physiological and clinical relevance of exercise-induced alterations in circulating S-Klotho. Furthermore, tissue-specific approaches, including arterio-venous balance measurements across exercising skeletal muscle, are needed to determine whether skeletal muscle contributes directly to the exercise-induced increase in circulating S-Klotho.

## Figures and Tables

**Table 1:** Anthropometry and exercise capacity.

	Women (*n* = 9)	Men (*n* = 9)	*p*
**ANTHROPOMETRICS**	Mean (SD)	Mean (SD)	
**Age**, years	26.7 (3.7)	26.3 (2.6)	0.83
**Body Weight**, kg	89.3 (12.5)	97.1 (11.9)	0.20
**Lean Body Mass**, kg	46.7 (5.0)	59.2 (6.6)	<0.01
**Body Fat Mass**, kg	39.7 (9.0)	34.6 (6.9)	0.20
**BMI**, kg·m^2^	30.4 (3.0)	30.2 (2.1)	0.89
**Lean Mass Index**, kg·m^2^	15.9 (0.6)	18.5 (1.4)	<0.01
**Body Fat**, %	44 (5)	35 (4)	0.01
**Visceral Fat**, g	984 (464)	1364 (747)	0.21
**Fat Mass Index**	13.5 (2.7)	10.8 (1.6)	0.02
**Waist Circumference**, cm	97.3 (8.7)	94.0 (8.4)	0.42
**CARDIORESPIRATORY FITNESS**	Mean (SD)	Mean (SD)	
**VO_2max_**, L·min^−1^	2.8 (0.4)	3.9 (0.6)	<0.01
**VO_2max_**, mL·min^−1^·kg^−1^	31.9 (2.6)	40.0 (5.8)	<0.01
**VO_2max_**, mL·min^−1^·kg LBM^−1^	60.8 (6.1)	65.2 (7.6)	0.19
BLOOD LIPIDS *	Median (Min–Max)	Median (Min–Max)	
**Total Cholesterol**, mmol·L^−1^	5.3 (4.5–6.3)	4.8 (1.2–4.9)	0.28 *
**TAG**, mmol·L^−1^	1.2 (0.6–3.4)	1.3 (0.6–1.0)	0.74 *
**Non-HDL**, mmol·L^−1^	3.9 (2.7–4.5)	3.4 (1.3–3.6)	0.74 *
**HDL**, mmol·L^−1^	1.7 (1.4–2.2)	1.4 (0.2–1.4)	0.01 *
INSULIN AND GLUCOSE *	Median (Min–Max)	Median (Min–Max)	
**HOMA-IR**	2.1 (1.8–6.1)	3.6 (1.9–10.2)	0.51 *
**HOMA-β**	145 (85–348)	125 (95–1931)	0.89 *
**fP-Glucose**, mmol·L^−1^	5.2 (4.9–5.8)	5.4 (4.1–6.3)	0.65 *
**fP-Insulin**, mIU·L^−1^	10.0 (8.0–27.0)	14.0 (8.0–56.0)	0.72 *
INFLAMMATION *	Median (Min–Max)	Median (Min–Max)	
**hsCRP**, mg·L^−1^	1.3 (0.2–8.5)	1.7 (1.0–13.5)	0.43 *

BMI = body mass index, TAG: triglyceride, Non-HDL: total cholesterol minus the high-density lipoprotein (HDL) cholesterol, fP = fasting serum value, hsCRP: high-sensitivity C-reactive protein, HOMA-IR: Homeostatic Model Assessment of Insulin Resistance, HOMA-β: Homeostasis Model Assessment, which estimates steady-state beta cell function. To test potential differences between men and women, unpaired *t*-test was used, with Mann–Whitney U test for blood variables *. Normal distribution was tested by Shapiro–Wilk Goodness-of-fit, *p* < 0.05.

**Table 3:** Comparative performance metrics for predictive models.

Model	R^2^	RASE	Selection Rationale
**PLS**	0.74	49	Selected: Optimal balance of fit and stability.
**FLS**	0.76	48	Rejected: High risk of overfitting in cross-validation.
**NB**	0.91	29	Rejected: Unstable performance on small sample size.
**GLM**	0.71	52	Rejected: Poor out-of-sample prediction.

Performance statistics for four models evaluating prediction of exercise-induced changes in serum Klotho. Included variables as predictors: visceral fat (g), waist circumference (cm), and Log-transformed C-reactive protein (CRP). R^2^ represents the coefficient of determination for the total sample (N = 18), RASE: Root Average Squared Error. The Partial Least Squares (PLS) model was selected for final reporting due to its superior stability and predictive validity during iterative cross-validation, whereas other tested models (Fit Least Squares, FLS; Neural Boosted, NB; Generalized Linear Model, GLM) were rejected due to high risk of overfitting given the sample size. Detailed cross-validation folds and training validation discrepancies for each model, and a model comparison, are provided in Supplementary Table S1 and File S1 to S6.

## Data Availability

The datasets generated and analyzed during the current study are not publicly available because they contain information that could compromise participant privacy and informed consent agreements. De-identified data may be made available by the corresponding author upon reasonable request and subject to approval by the relevant ethical authority.

## Supplementary Materials

The following supporting information can be downloaded at https://www.mdpi.com/article/10.3390/jcm15218313/s1: Table S1. Predictor screening by Bootstrap Forest (100 trees); File S1. Model Screening; File S2. Fit Partial Least Squares; File S3. Neural Boosted of Delta KLOTHO Pre-Post Maximal Exercise; File S4. FLS Delta KLOTHO Pre-Post Maximal Exercise; File S5. GLM Lasso of Delta KLOTHO Pre-Post Maximal Exercise; File S6. Model Comparison.
