# Supplementary material for: Acute Effects of Exhaustive Exercise on the Anti-Aging Protein S-Klotho in Young Females and Males with Obesity
Source: J Clin Med. 2026 Oct 26;15(21):8313. doi: 10.3390/jcm15218313 (PMC13764516; doi:10.3390/jcm15218313)

Model Screening for Delta KLOTHO Pre-Post Maximal Exercsie

Table: KLOTHO extended 20250324_CM   Response: Delta KLOTHO Pre-Post Maximal Exercsie

Details

Summary Across the Folds

			Test Set Folds					
Method	N Trials Folds	Sum Freq	Mean RSquare	StdDev RSquare		Mean RASE	StdDev RASE	
Partial Least Squares	5	3.6	0.5074	0.2968		64.041	11.995	
Neural Boosted	5	3.6	0.1451	0.7884		59.074	11.240	
Generalized Regression Lasso	5	3.6	-0.127	0.9393		73.596	25.716	
Fit Least Squares	5	3.6	-0.260	1.1695		66.211	26.623	

Select Dominant Run Selected Save Script Selected

Training

Method	N	RSquare	0...1	RASE	Fold
Neural Boosted	10	0.9507		22.597	5
Neural Boosted	13	0.9206		26.076	3
Neural Boosted	12	0.9061		22.596	4
Neural Boosted	13	0.9006		28.367	1
Fit Least Squares	10	0.8746		36.028	5
Neural Boosted	12	0.8531		37.211	2
Partial Least Squares	10	0.8367		41.122	5
Fit Least Squares	12	0.8075		32.350	4
Partial Least Squares	12	0.8075		32.350	4
Generalized Regression Lasso	12	0.8075		32.350	4
Fit Least Squares	12	0.8050		42.869	2
Fit Least Squares	13	0.7870		42.706	3
Generalized Regression Lasso	13	0.7864		42.766	3
Partial Least Squares	13	0.7842		42.991	3
Fit Least Squares	13	0.7588		44.195	1
Generalized Regression Lasso	12	0.7360		49.877	2
Partial Least Squares	13	0.6996		49.322	1
Partial Least Squares	12	0.6891		54.127	2
Generalized Regression Lasso	13	0.3090		74.803	1
Generalized Regression Lasso	10	0.1694		92.739	5

Select Dominant Run Selected Save Script Selected

Validation

Method		N	RSquare	0...1	RASE	Fold
Neural Boosted	†	2	0.9988		3.26	3
Neural Boosted	†	2	0.9711		24.82	5
Neural Boosted	†	3	0.9361		30.55	4
Neural Boosted	†	3	0.9304		31.89	2
Partial Least Squares	†	2	0.8979		30.04	3
Neural Boosted	†	2	0.8939		16.94	1
Generalized Regression Lasso	†	2	0.8786		32.75	3
Fit Least Squares		2	0.8766		33.02	3
Partial Least Squares	†	3	0.7586		59.37	2
Fit Least Squares		3	0.7299		62.81	4
Partial Least Squares	†	3	0.7299		62.81	4
Generalized Regression Lasso	†	3	0.7299		62.81	4
Generalized Regression Lasso	†	3	0.6743		68.97	2
Fit Least Squares		3	0.6169		74.80	2
Partial Least Squares	†	2	0.6005		92.28	5
Fit Least Squares		2	0.5290		100.20	5
Generalized Regression Lasso	†	2	0.0925		139.09	5
Partial Least Squares	†	2	-0.004		52.11	1
Fit Least Squares		2	-1.095		75.27	1
Generalized Regression Lasso	†	2	-1.169		76.58	1

† indicates that the method uses validation data to tune model.

Select Dominant Run Selected Save Script Selected

Test

Sum Freq and Sum Weight are suppressed when they are the same as N.

Actual by Predicted Plot

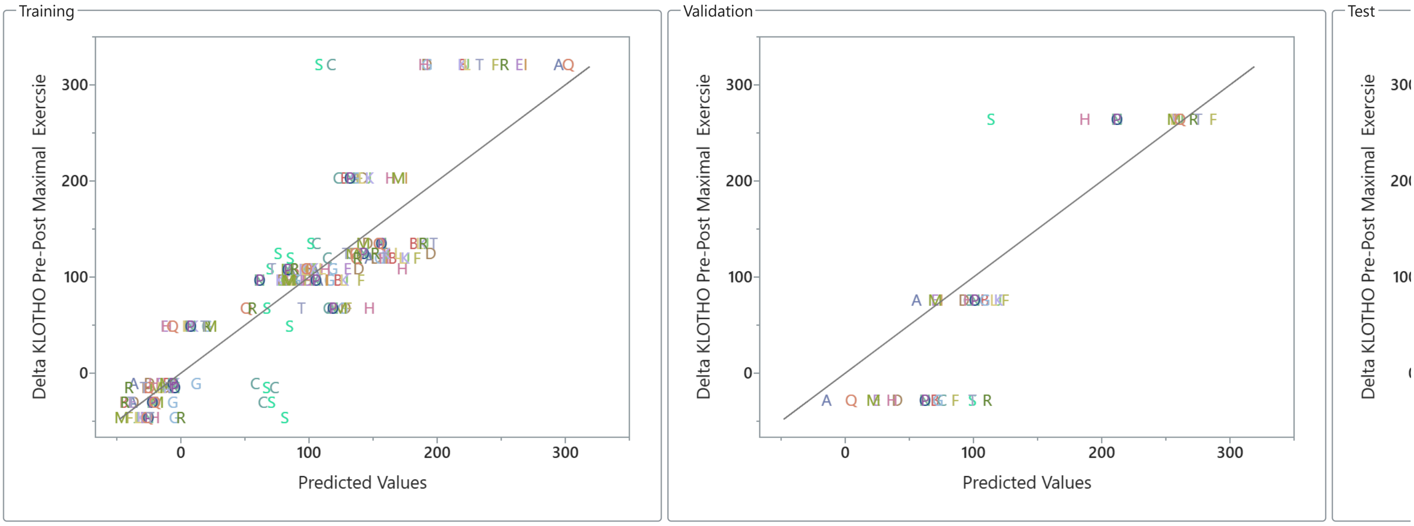

Prediction Profiler

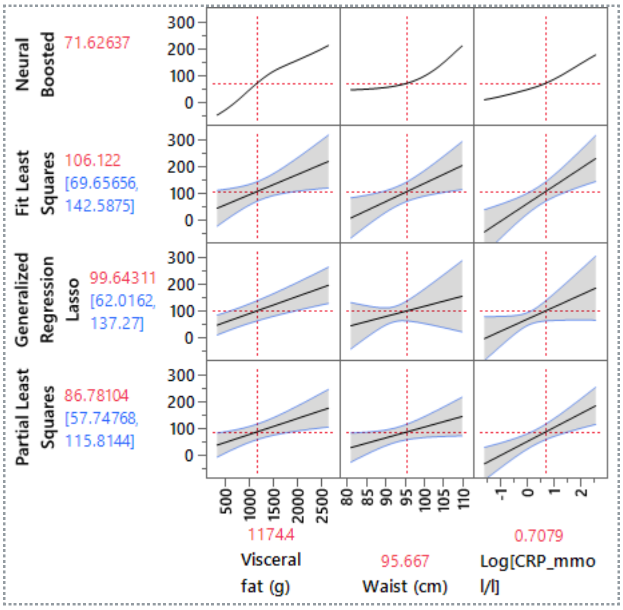

Supplement: Supplementary file 1 [file jcm-15-08313-s001.zip › File S1 - Model Screening.pdf]
