# Supplementary material for: Acute Effects of Exhaustive Exercise on the Anti-Aging Protein S-Klotho in Young Females and Males with Obesity
Source: J Clin Med. 2026 Oct 26;15(21):8313. doi: 10.3390/jcm15218313 (PMC13764516; doi:10.3390/jcm15218313)
Supplement: Supplementary file 1 [file jcm-15-08313-s001.zip › File S2 - Fit Partial Least Squares.pdf]

Partial Least Squares

Y response: Delta KLOTHO Pre-Post Maximal Exercsie
Validation Column: Validation Fold4

Model Launch

Method Specification
- NIPALS
- SIMPLS

Factor Specification
Number of Factors (1 to max=3) 3

Go

Model Comparison Summary

Method	SVD	Number of rows	Number of factors	Percent Variation Explained for Cumulative X	Percent Variation Explained for Cumulative Y	Number of VIP > 0.8
NIPALS	Fast	12	3	100	80.747613	2

Cross Validation with Validation Column and Method=NIPALS Using Fast SVD

Number of factors	Root Mean PRESS	van der Voet T^2	Prob > van der Voet T^2	Q^2	Cumulative Q^2	R^2X	Cumulative R^2X	R^2Y	Cumulative R^2Y
0	1.648964	0.967714	0.4960	-0.104159	-0.104159	0.000000	0.000000	0.000000	0.000000
1	1.086824	0.329410	0.7170	0.520346	0.520346	0.408550	0.408550	0.686253	0.686253
2	0.838910	0.023559	0.7330	0.714214	0.862922	0.358059	0.766609	0.094601	0.780855
3	0.815617	0.000000	1.0000	0.729864	0.962970	0.233391	1.000000	0.026621	0.807476

Root Mean PRESS Plot

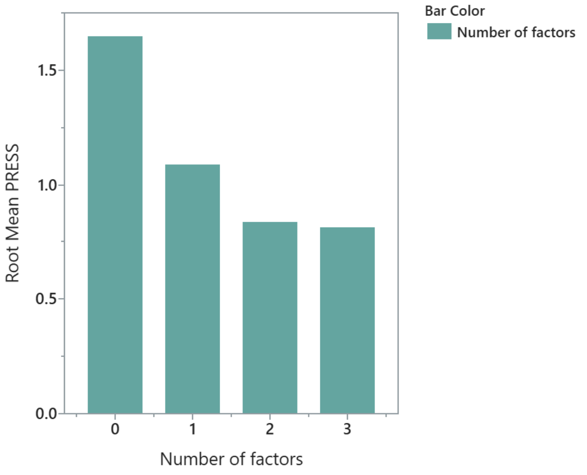

Note: The minimum root mean PRESS is 0.81562 and the minimizing number of factors is 3.

NIPALS Fit with 3 Factors Using Fast SVD

X-Y Scores Plots

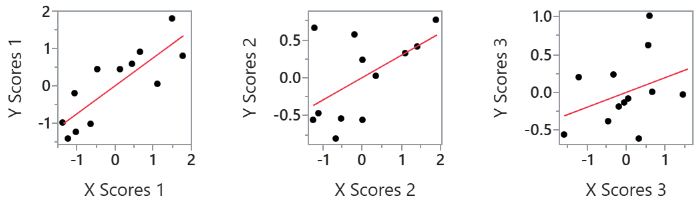

Percent Variation Explained

Number of factors	X Effects	Cumulative X
1	40.8550	40.855
2	35.8059	76.661
3	23.3391	100.000

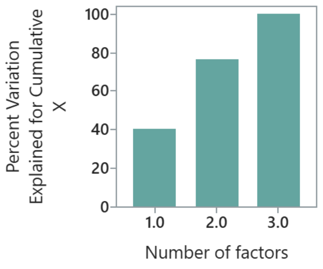

Y Responses	Cumulative Y
68.6253	68.6253
9.4601	78.0855
2.6621	80.7476

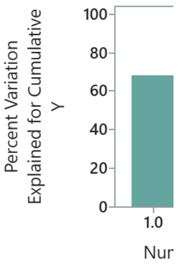

Crossvalidation

Source	Rsquare	RASE	Freq
Training Set	0.8075	32.3496	12
Validation Set	0.7299	62.8069	3
Test Set	0.7234	80.6200	3

Model Coefficients for Centered and Scaled Data

Term	Delta KLOTHO Pre-Post Maximal Exercsie
Intercept	0.0000
Visceral fat (g)	0.5082
Waist (cm)	0.4378
Log[CRP_mmol/l]	0.7907

Note: X variables have been standardized.

Model Coefficients for Original Data

Term	Delta KLOTHO Pre-Post Maximal Exercsie
Intercept	86.7822
Visceral fat (g)	37.5056
Waist (cm)	33.8075
Log[CRP_mmol/l]	56.2805

Note: X variables have been standardized.
