# Supplementary material for: Acute Effects of Exhaustive Exercise on the Anti-Aging Protein S-Klotho in Young Females and Males with Obesity
Source: J Clin Med. 2026 Oct 26;15(21):8313. doi: 10.3390/jcm15218313 (PMC13764516; doi:10.3390/jcm15218313)

Neural

Validation Column: Validation Fold5

Model Launch

Model NTanH(3)NBoost(19)

Training

Delta KLOTHO Pre-Post Maximal Exercsie

Measures	Value
RSquare	0.8565809
RASE	34.89693
Mean Abs Dev	25.898147
-LogLikelihood	59.656049
SSE	14613.548
Sum Freq	12

Value

Validation

Delta KLOTHO Pre-Post Maximal Exercsie

Measures	Value
RSquare	0.8195999
RASE	44.306813
Mean Abs Dev	33.188748
-LogLikelihood	31.260462
SSE	11778.562
Sum Freq	6

Prediction Profiler

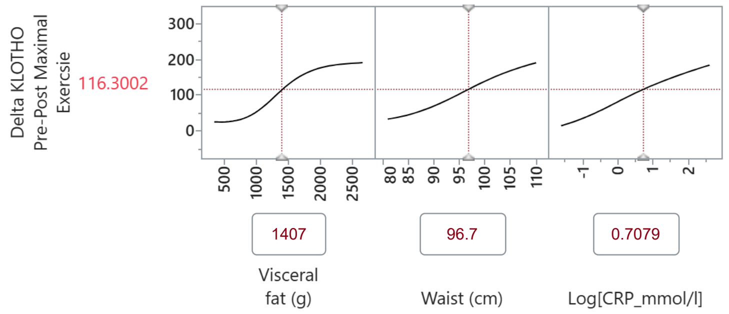

Animation Sequential

Actual by Predicted Plot

Training

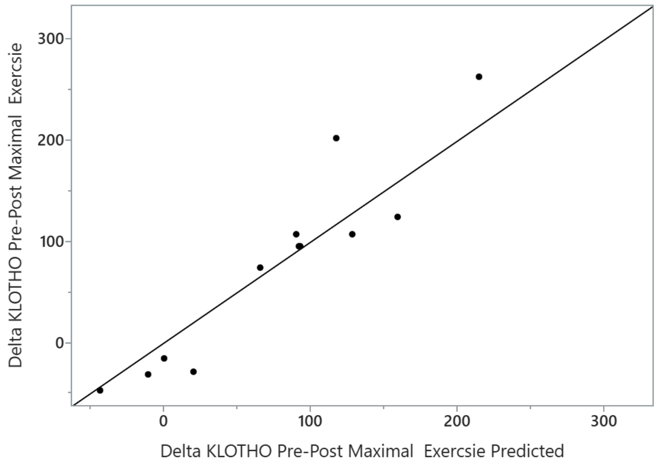

Validation

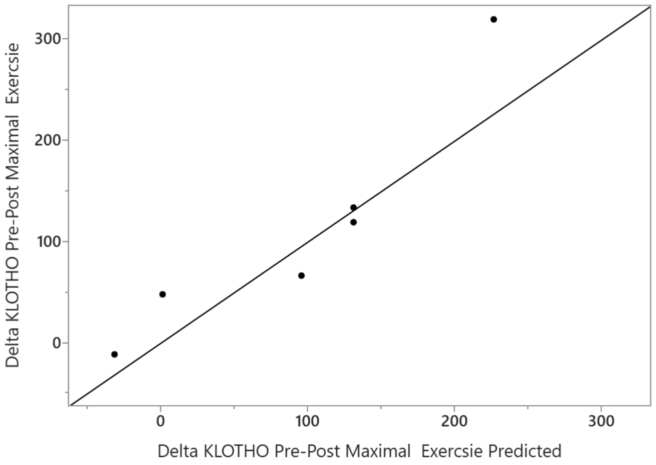

Supplement: Supplementary file 1 [file jcm-15-08313-s001.zip › File S3 - Neural Boosted of Delta KLOTHO Pre-Post Maximal Exercise.pdf]
