# Supplementary material for: Acute Effects of Exhaustive Exercise on the Anti-Aging Protein S-Klotho in Young Females and Males with Obesity
Source: J Clin Med. 2026 Oct 26;15(21):8313. doi: 10.3390/jcm15218313 (PMC13764516; doi:10.3390/jcm15218313)

Model Screening for Delta KLOTHO Pre-Post Maximal Exercsie

Table: KLOTHO extended 20250324_CM   Response: Delta KLOTHO Pre-Post Maximal Exercsie   Validation: Validation

Details

Response Delta KLOTHO Pre-Post Maximal Exercsie

Validation: Validation

Effect Summary

Source	Logworth	PValue
Visceral fat (g)	2.319	0.00480
Log[CRP_mmol/l]	2.172	0.00673
Waist (cm)	0.824	0.15002

Remove Add Edit Exclude ☐ FDR

Summary of Fit

RSquare	0.755957
RSquare Adj	0.67461
Root Mean Square Error	43.96972
Mean of Response	54.61538
Observations (or Sum Wgts)	13

Analysis of Variance

Source	DF	Sum of Squares	Mean Square	F Ratio
Model	3	53899.050	17966.4	9.2929
Error	9	17400.027	1933.3	**Prob > F**
C. Total	12	71299.077		0.0041*

Parameter Estimates

Term	Estimate	Std Error	t Ratio	Prob>|t|
Intercept	-303.2033	166.8852	-1.82	0.1026
Visceral fat (g)	0.0813337	0.021884	3.72	0.0048*
Waist (cm)	2.6915776	1.710395	1.57	0.1500
Log[CRP_mmol/l]	45.05178	12.87357	3.50	0.0067*

Effect Tests

Crossvalidation

Source	RSquare	RASE	Freq
Training Set	0.7560	36.585	13
Validation Set	0.2795	79.962	5

Effect Details

Training

Method	N	RSquare ↓	0...1	RASE
Fit Least Squares	13	0.7560		36.585

Select Dominant Run Selected Save Script Selected

Validation

Method	N	RSquare ↓	0...1	RASE
Fit Least Squares	5	0.2795		79.962

Select Dominant Run Selected Save Script Selected

Sum Freq and Sum Weight are suppressed when they are the same as N.

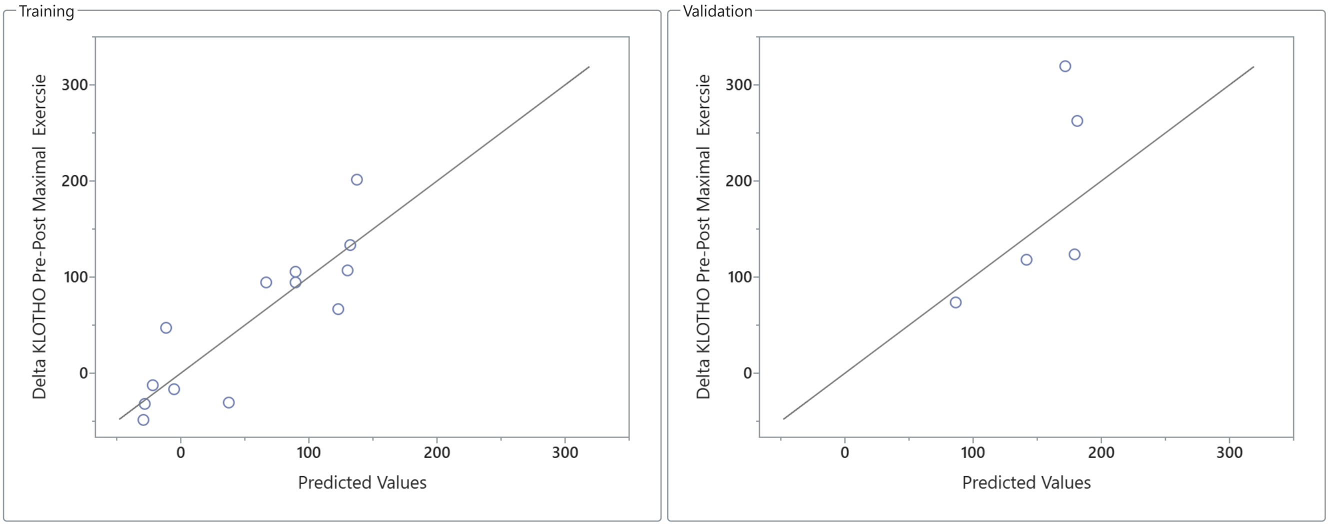

Prediction Profiler

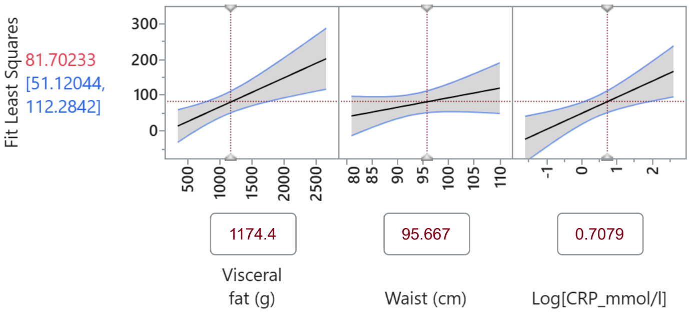

Supplement: Supplementary file 1 [file jcm-15-08313-s001.zip › File S4 - FLS Delta KLOTHO Pre-Post Maximal Exercise.pdf]
