# Supplementary material for: Acute Effects of Exhaustive Exercise on the Anti-Aging Protein S-Klotho in Young Females and Males with Obesity
Source: J Clin Med. 2026 Oct 26;15(21):8313. doi: 10.3390/jcm15218313 (PMC13764516; doi:10.3390/jcm15218313)

Model Screening for Delta KLOTHO Pre-Post Maximal Exercsie

Table: KLOTHO extended 20250324_CM   Response: Delta KLOTHO Pre-Post Maximal Exercsie   Validation: Validation

Details

Generalized Regression for Delta KLOTHO Pre-Post Maximal Exercsie

Model Comparison

Show	Response Distribution	Estimation Method	Validation Method	Nonzero Parameters	AICc	BIC	Generalized RSquare	Validation Generalized RSquare
☑	Normal	Lasso Estimation Method	Validation Column	5	149.05444	143.30776	0.7559572	0.2794835

Model Launch

Response Distribution
Normal

Estimation Method
Lasso
☐ Adaptive

Advanced Controls

Validation Method
Validation Column
☐ Early Stopping
Validation Column: Validation

Go

Normal Lasso with Validation Column

Model Summary

Response	Delta KLOTHO Pre-Post Maximal Exercsie
Distribution	Normal
Estimation Method	Lasso
Validation Method	Validation Column
Mean Model Link	Identity
Scale Model Link	Identity

Validation Column: Validation

Measure	Training	Validation
Number of rows	13	5
Sum of Frequencies	13	5
-LogLikelihood	65.241506	34.535645
Number of Parameters	5	5
BIC	143.30776	77.11848
AICc	149.05444	.
RSquare	0.7559572	0.2794835
RASE	36.585019	79.962359
Lambda Penalty	0	.

Estimation Details

Number of Grid Points	150
Minimum Penalty Fraction	0
Grid Scale	Square Root

Solution Path

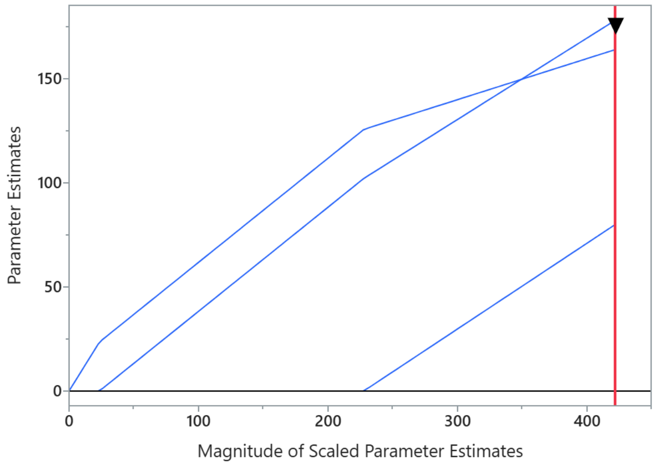

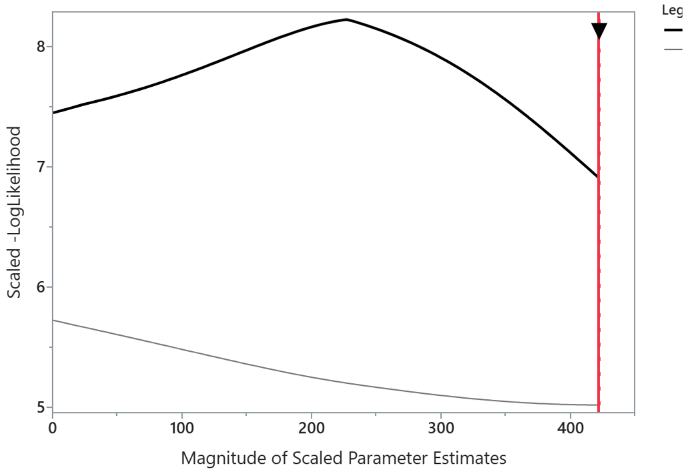

Parameter Estimates for Original Predictors

Term	Estimate	Std Error	Wald ChiSquare	Prob > ChiSquare	Lower 95%	Upper 95%
Intercept	-303.2033	95.411992	10.098616	0.0015*	-490.2073	-116.1992
Visceral fat (g)	0.0813337	0.0164391	24.478626	<.0001*	0.0491137	0.1135537
Waist (cm)	2.6915776	1.0704108	6.3228498	0.0119*	0.593611	4.7895441
Log[CRP_mmol/l]	45.05178	9.5653207	22.183232	<.0001*	26.304096	63.799464

Normal Distribution Parameters	Estimate	Std Error	Wald ChiSquare	Prob > ChiSquare	Lower 95%	Upper 95%
Scale	36.585019	6.3357888	33.343039	<.0001*	24.167101	49.002936

Training

Method	N	RSquare ↓	0...1	RASE
Generalized Regression Lasso	13	0.7560		36.585

Select Dominant Run Selected Save Script Selected

Validation

Method	N	RSquare ↓	0...1	RASE
Generalized Regression Lasso †	5	0.2795		79.962

† indicates that the method uses validation data to tune model.

Select Dominant Run Selected Save Script Selected

Sum Freq and Sum Weight are suppressed when they are the same as N.

Actual by Predicted Plot

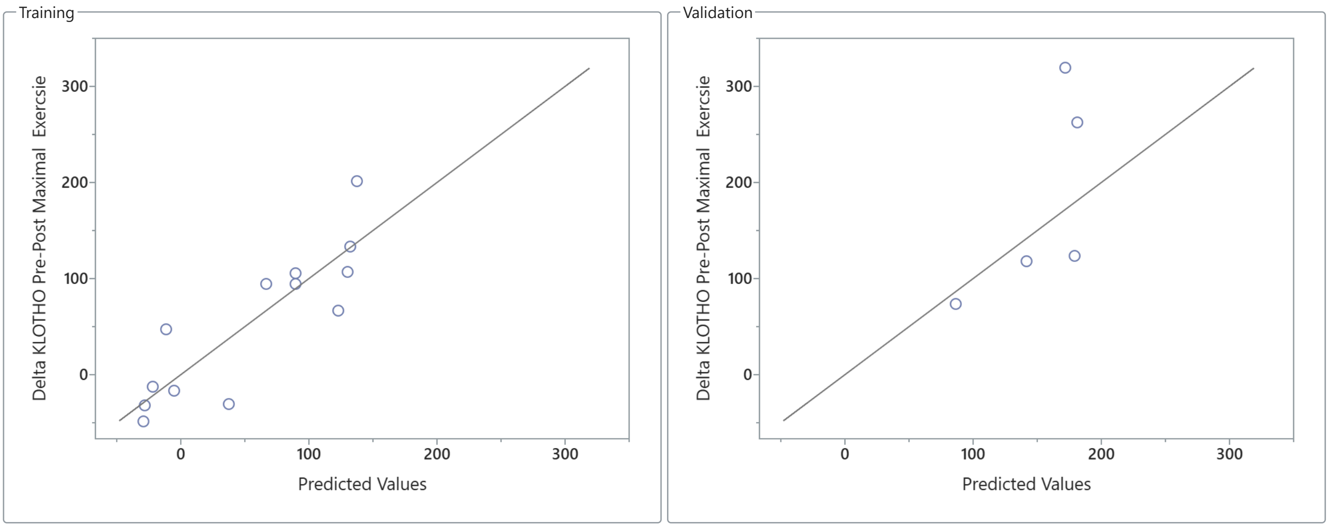

Prediction Profiler

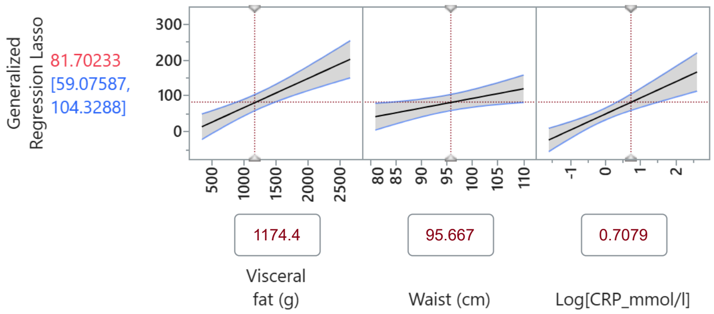

Supplement: Supplementary file 1 [file jcm-15-08313-s001.zip › File S5 - GLM Lasso of Delta KLOTHO Pre-Post Maximal Exercise.pdf]
