# Supplementary material for: Acute Effects of Exhaustive Exercise on the Anti-Aging Protein S-Klotho in Young Females and Males with Obesity
Source: J Clin Med. 2026 Oct 26;15(21):8313. doi: 10.3390/jcm15218313 (PMC13764516; doi:10.3390/jcm15218313)

Model Comparison

Predictors

Target	Predictors		
Delta KLOTHO Pre-Post Maximal Exercsie	GLM Prediction Formula	Fit Generalized Lasso	Validation: Validation
	PLS Pred Formula	Partial Least Squares	Validation: Validation Fold4
	BN at R2_93 Predicted	Neural	Validation: Validation

Measures of Fit for Delta KLOTHO Pre-Post Maximal Exercsie

Predictor	Creator	0...1	RSquare	RASE	AAE	Freq
GLM Prediction Formula	Fit Generalized Lasso		0.7125	52.371	37.957	18
PLS Pred Formula	Partial Least Squares		0.7444	49.380	37.360	18
BN at R2_93 Predicted	Neural		0.9129	28.830	18.867	18

Actual by Predicted Plot

Actual by Predicted Plot for Delta KLOTHO Pre-Post Maximal Exercsie

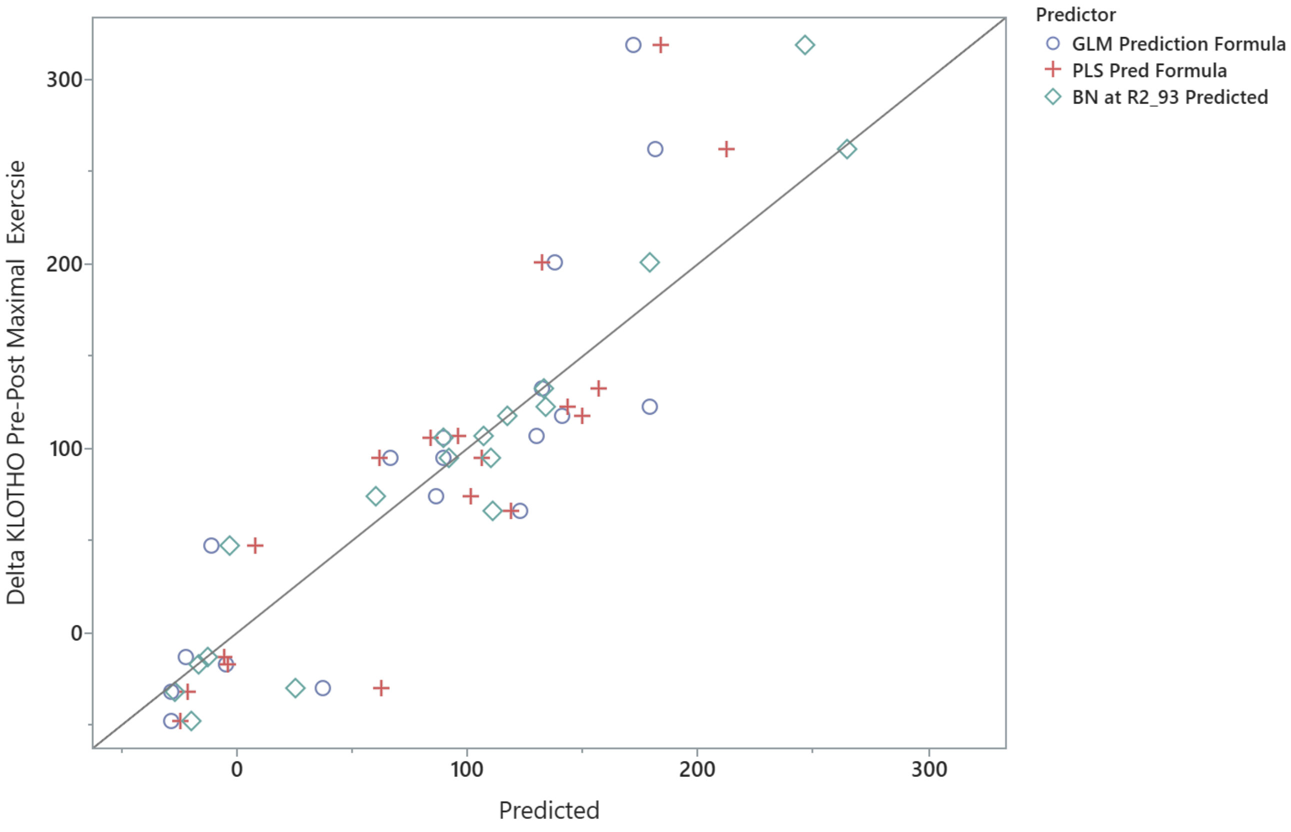

Prediction Profiler

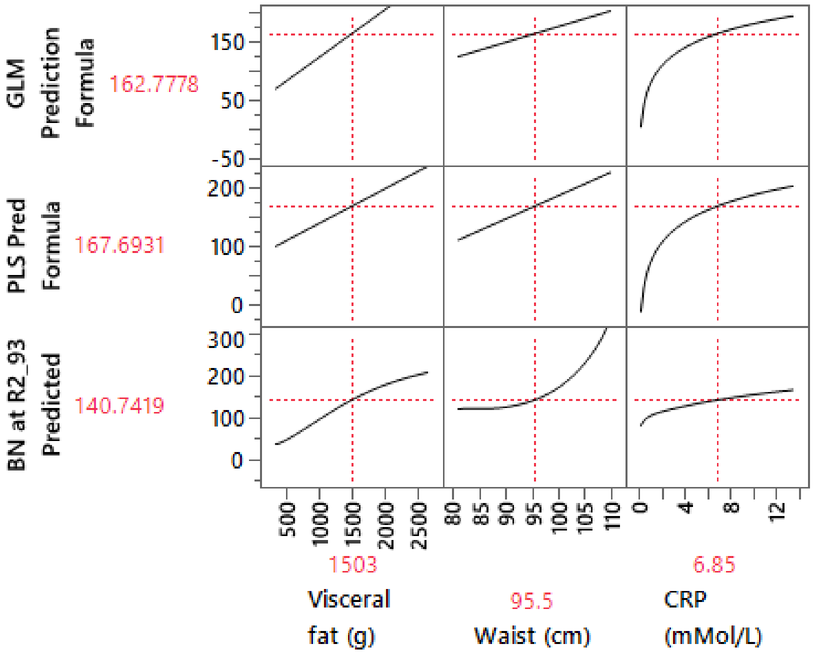

Supplement: Supplementary file 1 [file jcm-15-08313-s001.zip › File S6 - Model Comparison.pdf]
