# Supplementary material for: Acute Effects of Exhaustive Exercise on the Anti-Aging Protein S-Klotho in Young Females and Males with Obesity
Source: J Clin Med. 2026 Oct 26;15(21):8313. doi: 10.3390/jcm15218313 (PMC13764516; doi:10.3390/jcm15218313)
Supplement: Supplementary file 1 [file jcm-15-08313-s001.zip › Table S1.pdf]

Table S1. Predictor screening by Random Forest (100 trees)

Variable	Contribution	Portion
**Visceral fat** (g)*	20654	0.194
**Waist circumference** (cm)*	14111	0.133
**Body Weight** (kg)*	13246	0.125
**Klotho pre-exercise** ($pg \cdot L^{-1}$)*	10141	0.095
**Body Fat mass** (kg)	8489	0.080
**hsCRP** ($mmol \cdot L^{-1}$)*	8445	0.079
**TAG** ($mmol \cdot L^{-1}$)*	5401	0.051
**Total Cholesterol** ($mmol \cdot L^{-1}$)	4261	0.040
**Age** (years)	3235	0.030
**Lean Mass** (kg)	3022	0.028
**Non-HDL** ($mmol \cdot L^{-1}$)	2707	0.025
**Insulin** ($mIU \cdot L^{-1}$)	1950	0.018
**VO_{2max}** ($L \cdot min^{-1}$)	1729	0.016
**VO_{2max}** ($L \cdot min^{-1} \cdot$kg Lean Mass^{-1})	1660	0.016
**HDL** ($mmol \cdot L^{-1}$)	1528	0.014
**HOMA-IR**	1264	0.012
**VO_{2max}** ($L \cdot min^{-1} \cdot kg^{-1}$)	933	0.009
**Lean Mass Index**	766	0.007
**HOMA-b**	710	0.007
**Glucose** ($mmol \cdot L^{-1}$)	687	0.006
**Fat Mass Index**	633	0.006
**Sex**	368	0.003
**Body Fat** (%)	309	0.003

BMI = Body mass index, TAG: Triacylglycerides, Non-HDL: Total cholesterol minus the high-density lipoprotein (HDL) cholesterol, fP = Fasting plasma value, hsCRP: High-Sensitivity C-reactive protein, HOMA-IR: Homeostatic Model Assessment of Insulin Resistance, HOMA-β: Homeostasis Model Assessment estimates steady state beta cell function. *Used in models to predict S-Klotho. *Initial selection for models.
